# Selection at the Y Chromosome of the African Buffalo Driven by Rainfall

**DOI:** 10.1371/journal.pone.0001086

**Published:** 2007-10-31

**Authors:** Pim van Hooft, Barend J. Greyling, Herbert H. T. Prins, Wayne M. Getz, Anna E. Jolles, Armanda D. S. Bastos

**Affiliations:** 1 Mammal Research Institute, Department of Zoology and Entomology, University of Pretoria, Pretoria, South Africa; 2 Resource Ecology Group, Wageningen University, Wageningen, The Netherlands; 3 Department of Environmental Science Policy and Management, University of California at Berkeley, Berkeley, California, United States of America; 4 College of Veterinary Medicine and Department of Zoology, Oregon State, University, Corvallis, Oregon, United States of America; The University of New South Wales, Australia

## Abstract

Selection coefficients at the mammalian Y chromosome typically do not deviate strongly from neutrality. Here we show that strong balancing selection, maintaining intermediate frequencies of DNA sequence variants, acts on the Y chromosome in two populations of African buffalo (*Syncerus caffer*). Significant correlations exist between sequence variant frequencies and annual rainfall in the years before conception, with five- to eightfold frequency changes over short time periods. Annual rainfall variation drives the balancing of sequence variant frequencies, probably by affecting parental condition. We conclude that sequence variants confer improved male reproductive success after either dry or wet years, making the population composition and dynamics very sensitive to climate change. The mammalian Y chromosome, interacting with ecological processes, may affect male reproductive success much more strongly than previously thought.

## Introduction

The use of Y chromosomal genetic markers has made important contributions to studies of evolution, male-specific demography, and sexual selection in humans [1]–[4]. As with the maternally inherited mitochondrial DNA, the Y chromosome is a single, haploid non-recombining unit, except for a small pseudoautosomal region. Both positive and negative selection act on the mammalian Y chromosome [2], [3], and effects from balancing selection are considered unlikely for a haploid chromosome [3]. Most contemporary selective effects appear to be so small that some authors regard the Y chromosome as essentially neutral [3].

Recently, a large number (38) of polymorphic Y chromosomal microsatellites (tandem repeats of DNA sequence units that are one to five nucleotides in length) have been developed for cattle (*Bos taurus*) [5], [6]. We selected three of these to look for signs of selection in the African buffalo (*Syncerus caffer*).

African buffalo are polygamous and live in herds of up to 1,600 individuals. Bulls only peripherally associate with the herd, occurring also as solitary individuals and in small bachelor herds [7]. We studied the populations in Kruger National Park (KNP) and Hluhluwe-iMfolozi Park (HiP) in South Africa. The two populations differ in size by an order of magnitude (KNP: 31,000, HiP: 3,000) [8], [9]. They also differ in social organization, with large unstable herds and overlapping home-ranges in KNP versus small, fairly stable herds in HiP with home ranges that generally do not overlap [10], [11].

Selection may act on one of the many Y chromosomal genes that play a role in spermatogenesis [1], [5], [12], [13], a complex process that greatly influences male reproductive success. We hypothesized that at the phenotypic level, selection acts on body condition because sperm production is costly and probably traded off against other life-history traits [14], [15], and that this selection influences Y chromosomal gene frequencies among male offspring. Body condition is related to the quality and availability of food resources, which are influenced by the amount of rainfall [16]. Annual rainfall in KNP and HiP varies between 244/397 and 1077/1226 mm (KNP/HiP, period 1979–2004; Figure S1). Variable rainfall is one of the driving forces in African savanna ecosystems, affecting ungulate population dynamics through its effect on habitat conditions, food availability and predator-prey interactions [17]–[21]. Here we used a temporal correlation analysis to identify selective effects of rainfall on the Y chromosome.

## Results

We tested the hypothesis of selective effects of rainfall on the Y chromosome in both a forward and backward stepwise (conditional) logistic regression. The regression functions involved DNA sequence variants as a dependent variable and six independent variables: year of birth, annual rainfall in the year of birth, as well as in each of the three years before birth, and locality. Although rainfall in the year of birth occurs after conception, it was included in the regression because there is a negative autocorrelation in annual rainfall with a time lag of two years (period 1983–2004/1981–2002, KNP: *P* = 0.047, HiP: *P* = 0.046; Figures S2 and S3), which may result in opposite correlations with the second year before birth.

**Figure 1:**
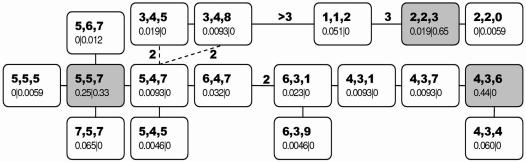
Minimum spanning network of Y chromosomal haplogroups. The minimum spanning network was constructed manually by minimization of the number of (stepwise) mutations between haplotypes with preference given to the mutation of the most polymorphic haplotype when alternative connections were possible. The first row of numbers refers to the different haplotypes at, respectively, microsatellites UMN0304, UMN113, and INRA189. The second row of numbers refers to the haplogroup frequencies in KNP and HiP respectively. The minimum number of mutations is indicated if this is greater than one, roughly corresponding to the length of the connecting line. Grey haplogroups: the three most frequent haplogroups. Dashed lines: alternative connections.

**Figure 2:**
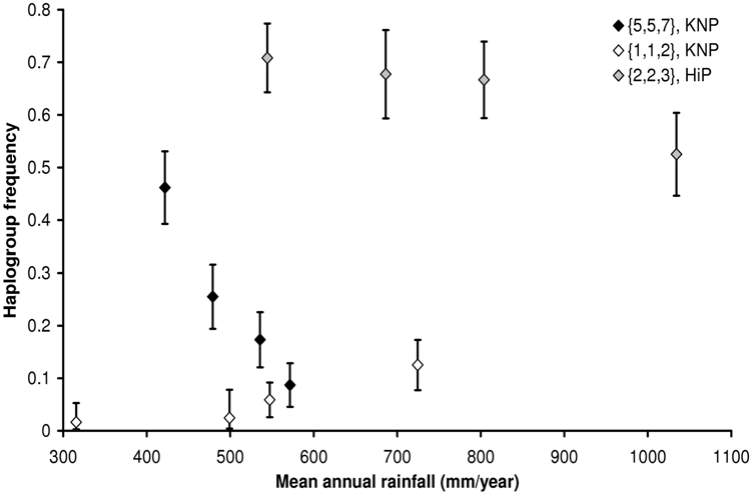
Correlation between haplogroup frequency and mean annual rainfall. {*n*_1_, *n*_2_, *n*_3_}: haplogroup. Black diamonds: mean annual rainfall in the three years before birth. Open diamonds: rainfall in the second year before birth. Grey diamonds: rainfall in the year of birth. Error bars: standard error (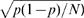), except for the two lowest data points of haplogroup {1,1,2} in which the haplogroup was observed only once. Here they correspond to a chance ≥84% of sampling ≤1 (positive error bar) or ≥1 haplogroup (negative error bar). Sample sizes were equalized as much as possible.

**Figure 3:**
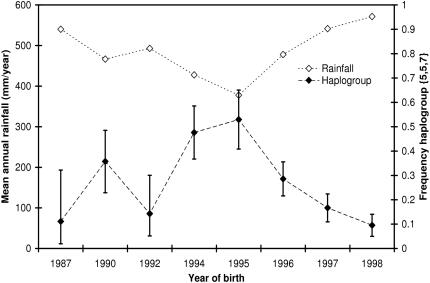
Negative correlation between frequency haplogroup {5,5,7} in KNP and rainfall in the three years before birth. Error bars: standard error (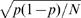), except for the two lowest data points in which the haplogroup was observed only once or twice. Here they correspond to a chance ≥84% of sampling ≤1 or 2 (positive error bar) or ≥1 or 2 haplogroups (negative error bar). Years of birth 1987, 1990, 1992 and 1994 consist of pooled years because of the small sample size (with the *x* axis corresponding to the average year of birth). Years of birth were pooled with similar amounts of rainfall in the preceding three years (difference<69 mm/year).

We observed 15 haplogroups (groups of tightly linked haplotypes, with each haplogroup representing a DNA sequence variant) in KNP with gene diversity *Ĥ* = 0.737±0.024 (standard error) and five in HiP with *Ĥ* = 0.475±0.026. Similar values for *Ĥ* were observed at the herd level (KNP: 0.729; HiP: 0.478). The total number of haplogroups was 18, with two being found in both populations ({2,2,3} and {5,5,7}). The minimum spanning (parsimony) network (MSN) in Figure 1 depicts the relationships among the haplogroups. With six haplogroups, significant or near-significant relationships were observed with one or more of the parameters in the regression model (Table 1, Figures 2 and 3, Table S1). Rainfall appeared to have a very strong effect, with haplogroup frequencies differing by as much as five- to eightfold between dry and wet years (Figures 2 and 3, haplogroups {1,1,2}and { 5,5,7} in KNP).

**Table 1:** *P* values of the parameters in the logistic regression model for haplogroup frequencies.

Parameter	KNP {6,4,7}	KNP {4,3,6}	KNP {5,5,7}^1^	KNP {7,5,7}	KNP {1,1,2}	HiP {2,2,3}^2^
**Year of birth**	0.040^−^	0.044^+^	-	-	0.046^−^	0.0090^+^
**Rainfall 3 y before birth**	-	-	0.019^−^	-	-	-
**Rainfall 2 y before birth**	-	-	0.0059^−^	-	0.011^+^	-
**Rainfall 1 y before birth**	-	-	0.0032^−^	-	-	-
**Rainfall year of birth**	-	-	-	0.053^+^	-	0.011^−^
**Locality**	-	0.0041^−^	0.00060^+^	-	-	0.025

-: *P*>0.054, {*n*_1_, *n*_2_, *n*_3_}: haplogroup

The direction of correlation is indicated in superscript. A positive correlation with locality is associated with high frequencies in southern KNP.

1: Correlation with mean annual rainfall across the three years before birth: *P*<0.0001.

2: Frequency haplogroup {5,5,7} in HiP ≈ 1-frequency haplogroup {2,2,3}

The correlations with year of birth and locality, independent of the temporal variation in rainfall, may be associated with additional factors influencing body condition, such as bovine tuberculosis prevalence, which has increased in both populations since the late 1980s and which in KNP shows a spatial correlation with haplogroups {4,3,6} and {5,5,7} [8], [11], [31], [32].

## Discussion

Because the gestation period in buffalo is±340 days [7]—almost one year—correlations with rainfall in each of the three years before birth can be equally regarded as correlations with rainfall in the year of and in each of the two years before conception. This observation indicates that selection is associated with the body condition of one or both parents during mating and with the development of body condition in the two preceding years. The significant correlations observed between rainfall in the year of birth and haplogroups {2,2,3} and {7,5,7} (Table 1) means that we cannot rule out selection after conception. However, these correlations are opposite those observed between rainfall in the second year before birth and the closely related haplogroups {1,1,2} and {5,5,7} (Table 1), which are neighboring haplogroups {2,2,3} and {7,5,7} in the MSN, indicating an artifact of the negative autocorrelation in annual rainfall. The focal gene under selection appears to consist of at least three alleles in linkage with the three most frequent haplogroups ({2,2,3}, {4,3,6} and {5,5,7}), considering the variation in correlations (Table 1) and the relationships among the different haplogroups in the MSN. We conclude that in African buffalo, Y chromosome sequence variants drastically affect male reproductive success, conferring on their bearer improved reproductive success after either dry or wet years. Yearly variation in annual rainfall thus has a direct and immediate effect on the population composition and dynamics.

A major effect of the Y chromosome on the genetic variation for reproductive success has not been observed in mammals before, and the only other example we can find is in *Drosophila melanogaster* [12]. Because positive (directional) selection and genetic drift would quickly have eroded genetic variation, the haplogroup frequencies must be maintained at intermediate levels over longer time periods by balancing selection driven by variation in rainfall. This idea is supported by the relatively large genetic differences among the three most frequent haplogroups in the MSN. It has been hypothesized that selective sweeps (positive selection) and negative selection influence the evolution of the Y chromosome, which may explain its relatively low genetic variability [22]. The presence of balancing selection, which increases genetic variability, shows that the evolution of the Y chromosome is more complex than previously thought and that conclusions from genetic studies that assume neutrality may need to be re-evaluated.

A possible explanation for our observations is that selection is related to investment in sperm production, which is traded off against body condition, an important factor in mating success [14]. Alternatively, selection may be related to fertilization success, without a trade-off with mating success, as this is typically a polygenic character with limited effects of each contributing gene [23]. We think that post-zygotic selection is unlikely as it relies on sex-biased embryonic mortality, which is constrained by the extent of sex dimorphism and its variance, as well as by the maximum possible reduction in blastocyst numbers [24]. Such a constraint is at odds with the strong yearly variation in haplogroup frequencies observed here. Furthermore, in the closely related American bison (*Bison bison*), birth sex ratio variation has been observed in the absence of the reduction of blastocyst numbers or of spontaneous abortion [24].

Gene (haplogroup) diversity may be maintained by spatiotemporal fluctuations in selection pressure due to environmental heterogeneity [25] or, when selection is related to fertilization success, by post-copulatory sexual selection [15], [26]. Gene fixation can then be prevented by variation in the female population's preference for specific spermatozoa (cryptic female choice) [27] or frequency-dependent sperm competition, with the fitness pay-offs being related to the number of fertile bulls willing to mate or females in estrus, both under the influence of body condition [28].

To the best of our knowledge, this study constitutes the strongest case yet for an association between a single environmental driver and allele (haplogroup) frequencies in a mammalian species. The strong relationship with male reproductive success makes the population composition and dynamics very sensitive to changes in rainfall pattern due to climate change [29], which may ultimately affect population viability. Considering the high degree of chromosomal conservation [1], we expect that Y chromosomal balancing selection will be identified in more mammalian species. Additional studies of Y chromosomal genetic variation in species that vary with respect to social structure and mating behavior are needed to provide more insights in the underlying physiological and ecological mechanisms.

## Materials and Methods

This study was performed in two South African conservation parks, with KNP (22°–25°S, 31°–32°E) comprising 20,000 km^2^ and 31,000 buffalo [9] (1.6/km^2^) and HiP (28°S, 31°–32°E) comprising 960 km^2^ and 3,000 buffalo [8] (3.6/km^2^). Blood samples were collected from tranquillized males from 31 herds in KNP from September to November 1998 [30], [31], and from 13 herds in HiP from May to June 2002, April 2003 and September to October 2004. The sample size for KNP was 216 and for HiP 170 individuals. DNA was isolated using standard extraction protocols. The age of the buffalo was estimated in years on the basis of dental wear patterns, number of erupted incisor teeth, body size, and horn development [8], [30]. The age estimates varied between 0 and 15 years. No accurate age estimates could be obtained for 15 individuals from KNP and nine individuals from HiP, reducing the sample size for most statistical analyses to respectively 201 and 161 individuals.

We tested 37 (out of 38, excluding microsatellite UMN0705 as it did not show clear loci in cattle) polymorphic Y chromosomal microsatellites from cattle [5], [6] for PCR amplification and polymorphism on a panel of male and female African buffalo. For the first round of amplification, forward primers with a 5′ M13 tail (TGT AAA ACG ACG GCC AGT) were used. Of these, 34 microsatellites could be amplified, and 18 were subsequently analyzed for polymorphism with a single fluorescently labeled M13 primer, which replaced the M13 extended forward primers in a second amplification round. Three microsatellites, UMN0304, UMN1113, and INRA189, were selected for further analyses on the basis of male-specificity, localization on the Y-specific region in cattle [5], polymorphism, ease of haplotype scoring, and possibility of multiplexing the PCR reactions and co-loading them on a DNA sequencer. Multiplex PCR reactions were performed with the Qiagen Multiplex PCR kit following the manufacturer's instructions. Reactions were performed in 28 annealing cycles of 57°C in 7 µl volumes containing 0.5 µl of DNA template, and between 0.1 and 0.3 µM of each primer set, with each forward primer having a unique fluorescent label. The PCR products were genotyped on an ABI 3100 DNA sequencer (Applied Biosystems) and analyzed with GeneMapper® software (Applied Biosystems). Each unique haplogroup was subsequently confirmed in monoplex PCR reactions.

Unlike autosomal microsatellites, many bovine Y-chromosomal microsatellites occur as multicopies [6], which together constitute a haplotype. We observed six, seven, and ten haplotypes at the UMN1113, UMN0304, and INRA189 loci, respectively. As there is no recombination, the haplotypes can be combined into 18 haplogroups (two haplogroups were found in both populations). UMN0304 consists of the following polymorphic loci (number of nucleotides): 213-223-225 (1), 215-225 (2), 205-213-223 (3), 205-215 (4), 205-213-221 (5), 205-215-221 (6), and 213-221 (7). UM1113 consists of the following polymorphic loci: 131-133 (1), 133 (2), 131-159 (3), 131-157 (4), 131-155 (5), and 131-153 (6). INRA189 consists of the following polymorphic loci: 147-151-156-158-162 (0), 149-158 (1), 147-151-156-164 (2), 147-151-156-162 (3), 160 (4), 147-160 (5), 158 (6), 147-158 (7), 147-156 (8), and 149-151 (9). Thus, taking all three loci into account, a haplogroup can be written as {*n*_1_, *n*_2_, *n*_3_}, where *n*_1_ = 1,…,6, *n*_2_ = 1,…,7, and *n*_3_ = 0,…,9).

Monthly rainfall data from 1979 to 2004 were averaged across 14 rainfall stations from the South African Weather Service (SAWS) in KNP and across eight rainfall stations from the KwaZulu-Natal Wildlife organization in HiP. The “rainfall years” run from September to August because the wet season falls between October and March.

Unbiased estimates of gene diversity (*Ĥ*, probability of randomly sampling two different haplogroups, equivalent to the expected heterozygosity for diploid data) and its standard error were obtained with Arlequin 3.0. We used both a forward and backward stepwise (conditional) logistic regression model with SPSS 12.0.1 for analyzing relationships with haplogroup frequencies. A separate regression was performed for each haplogroup. The resulting *P* values were appropriate for each individual regression, although the different regressions for a single population were obviously not independent. In KNP, locality was treated as a continuous covariate consisting of latitudinal coordinates (herds sampled between 22.3° and 25.5°), and in HiP, as a categorical covariate (coordinates from some herds were not available) consisting of the five management sections: Makhamisa, Manzibomvu, Masinda, Mbuzane, and Nqumeni (with herds sampled from each). Rainfall data were analyzed for autocorrelation with time lags of one, two, and three years by estimation of the significance of the correlation between rainfall in year x and rainfall in year x minus 1, 2, or 3 years, using the Spearman rank test.

## Supporting Information

Table S1 (0.05 MB DOC)

Figure S1 Annual rainfall (Sept.-Aug.) in KNP and HiP.
(0.37 MB TIF)

Figure S2 Negative autocorrelation in annual rainfall (period 1983–2004/1981–2002) with a time lag of two years.
(0.32 MB TIF)

Figure S3 Negative autocorrelation in annual rainfall between year of birth and second year before birth among the sampled individuals. HiP: Spearman rank correlation: *P* = 0.17, but *P* = 0.0045 when excluding six outlier samples (grey, 3 data points, 3.7% of all samples). KNP: Spearman rank correlation: *P* = 0.23, but *P* = 0.047 when rainfall in year of birth ≤ 348 mm/year is excluded (14% of all samples). A large fraction (86–96%) of the samples is characterized by a negative autocorrelation, which can affect the resulting *P* values in the logistic regression model, i.e., giving significant values in the year of birth rather than the second year before birth.
(0.33 MB TIF)
