# Supplementary material for: Selection at the Y Chromosome of the African Buffalo Driven by Rainfall
Source: PLoS One. 2007 Oct 31;2(10):e1086. doi: 10.1371/journal.pone.0001086 (PMC2034602; doi:10.1371/journal.pone.0001086)
Supplement: Table S1 — (0.05 MB DOC) [file pone.0001086.s001.doc]

Table S1. Estimates and standard errors of the parameters in the logistic regression model for haplogroup frequencies.

| ***Parameter*** | ***KNP***  ***{6,4,7}*** | ***KNP***  ***{4,3,6}*** | ***KNP***  ***{5,5,7}*** | ***KNP***  ***{7,5,7}*** | ***KNP***  ***{1,1,2}*** | ***HiP***  ***{2,2,3}*** |
| --- | --- | --- | --- | --- | --- | --- |
| **Year of birth** | -0.20±0.10 | 0.102±0.051 | - | - | -0.152±0.077 | 0.121±0.046 |
| **Rainfall 3 y b. birth** | - | - | -0.050±0.021 | - | - | - |
| **Rainfall 2 y b. birth** | - | - | -0.043±0.016 | - | 0.081±0.032 | - |
| **Rainfall 1 y b. birth** | - | - | -0.057±0.019 | - | - | - |
| **Rainfall year of birth** | - | - | - | 0.042±0.022 | - | -0.029±0.012 |
| **Locality** | - | -0.42±0.15 | 0.78±0.23 | - | - | *1 |
| **Intercept** | 15.4±9.0 | 0.2±6.1 | -14.1±5.7 | -4.4±1.0 | 7.7±7.0 | -9.8±4.6 |

{*n*_1_, *n*_2_, *n*_3_}: haplogroup, -: no significant relationship (*P* > 0.054), *1: categorical parameter with different values when entered at different steps in the logistic regression model.
