# Supplementary figures and images for: Selection at the Y Chromosome of the African Buffalo Driven by Rainfall
Source: PLoS One. 2007 Oct 31;2(10):e1086. doi: 10.1371/journal.pone.0001086 (PMC2034602; doi:10.1371/journal.pone.0001086)

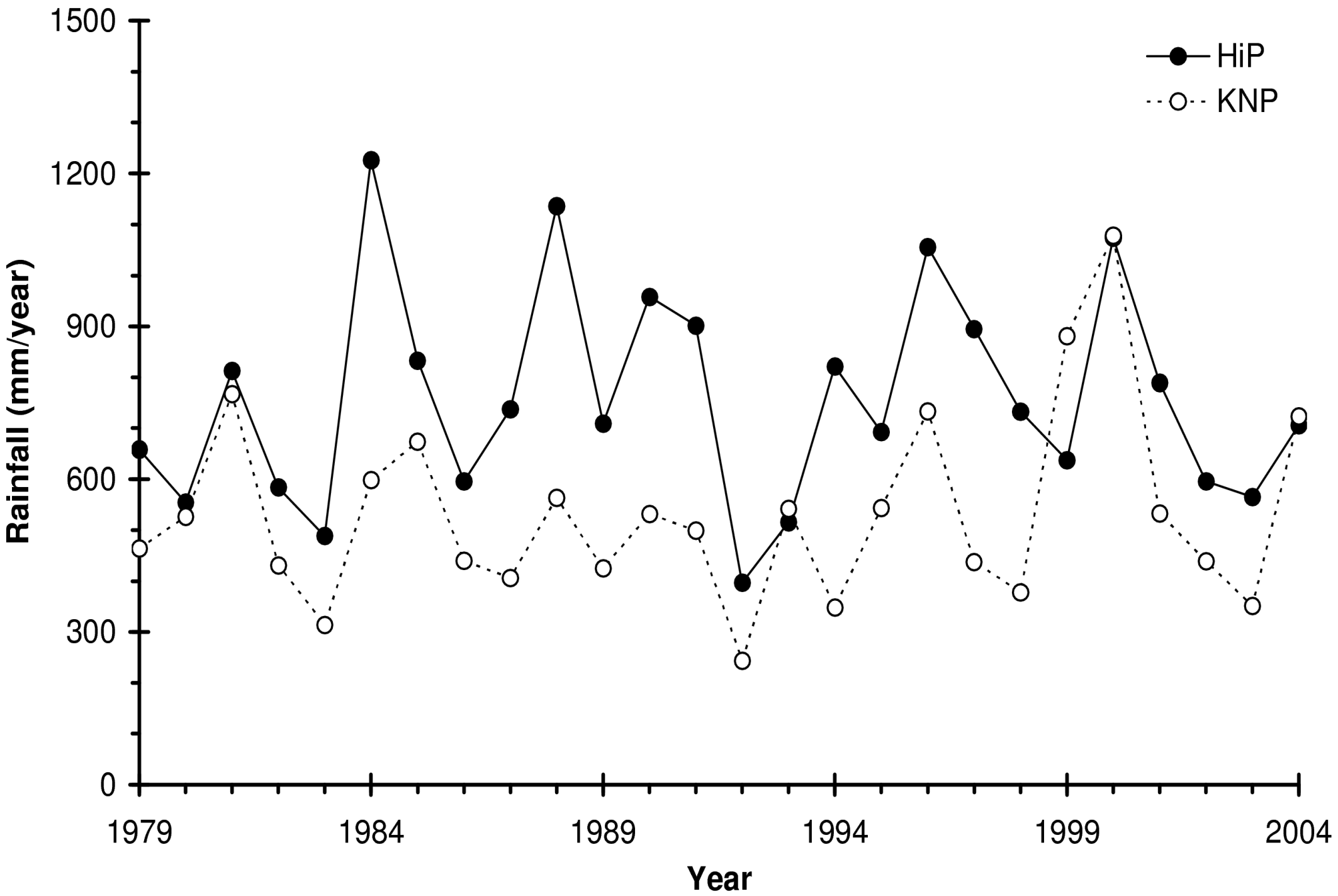

Supplement: Figure S1 — Annual rainfall (Sept.-Aug.) in KNP and HiP. (0.37 MB TIF) [file pone.0001086.s002.tif]

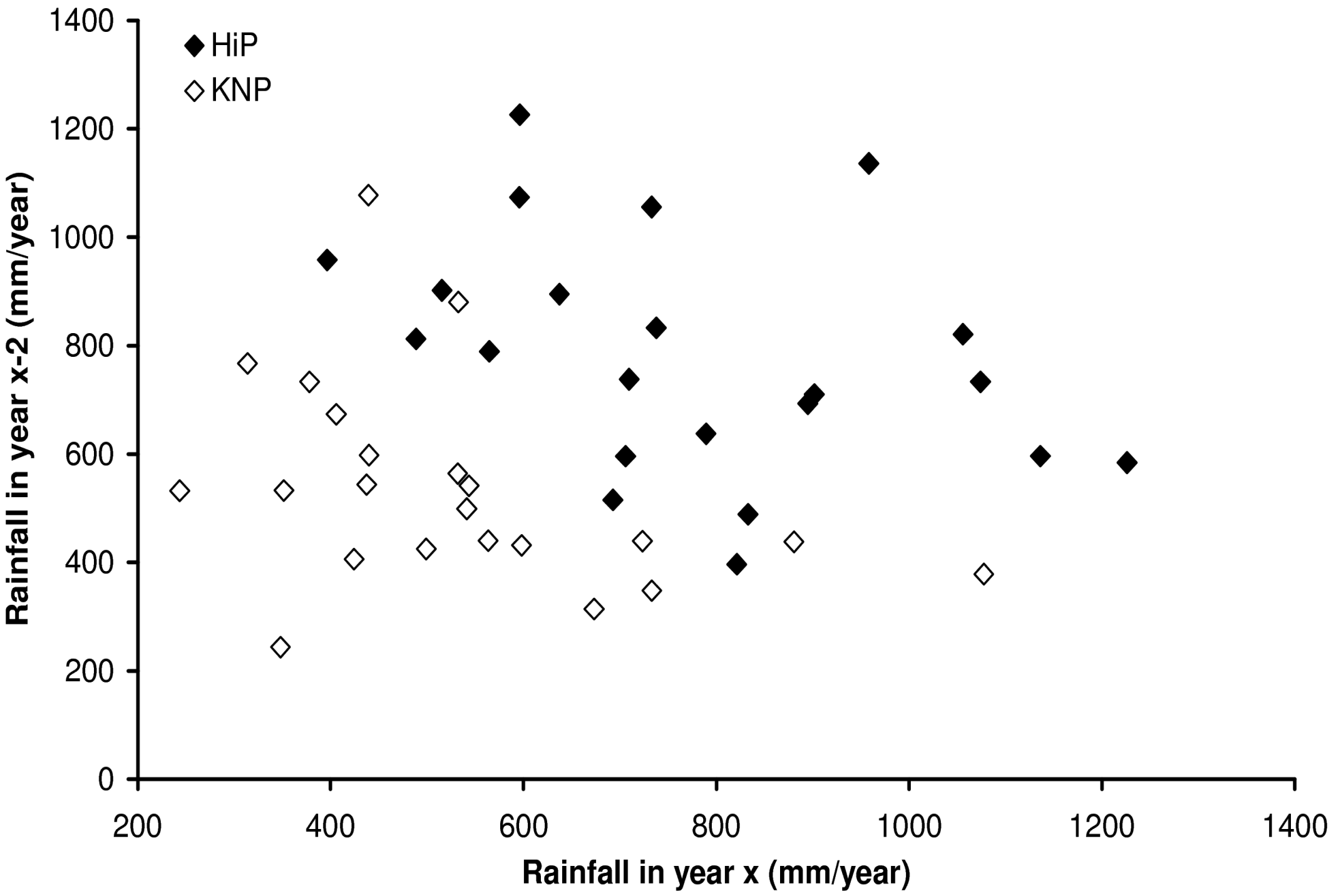

Supplement: Figure S2 — Negative autocorrelation in annual rainfall (period 1983–2004/1981–2002) with a time lag of two years. (0.32 MB TIF) [file pone.0001086.s003.tif]

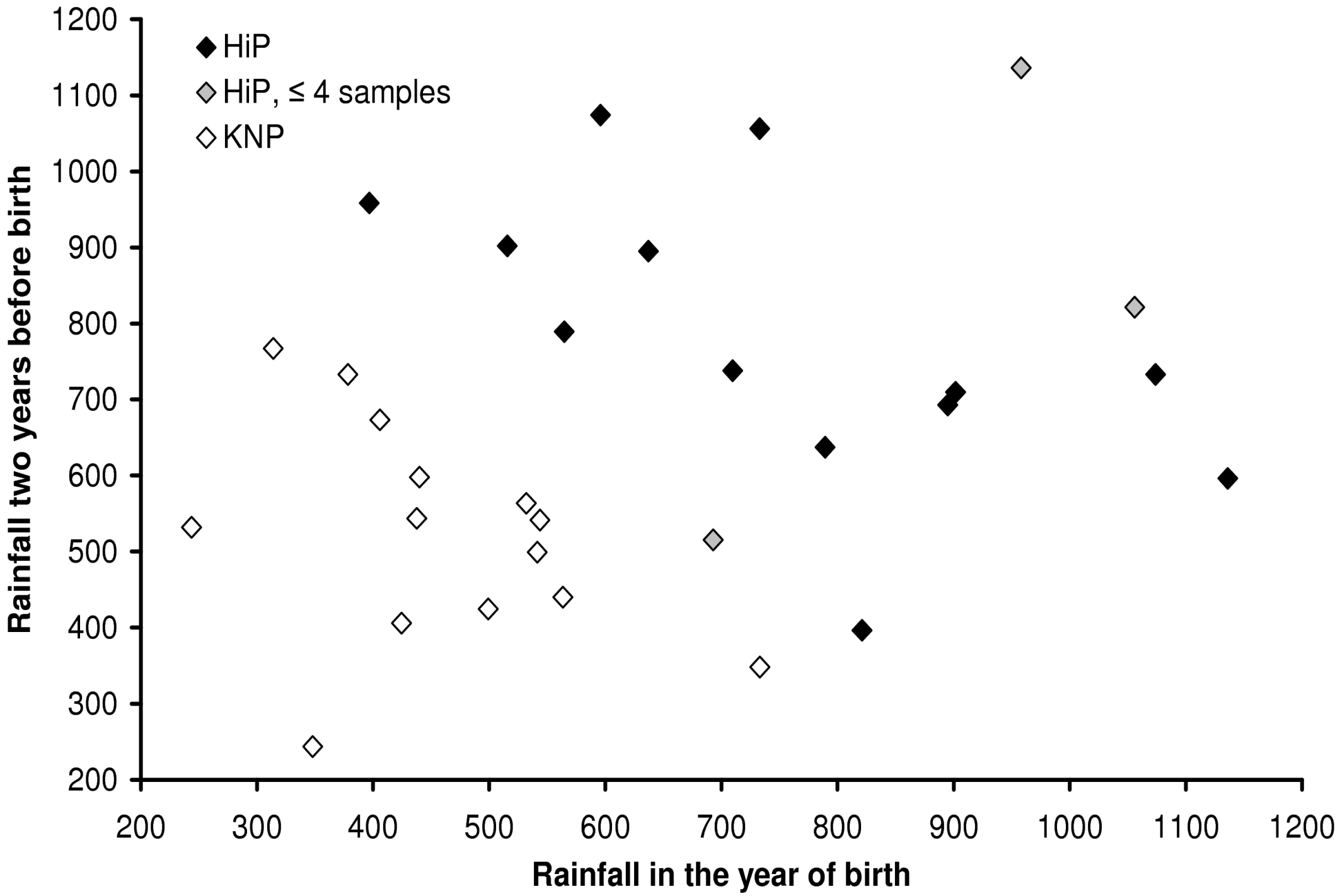

Supplement: Figure S3 — Negative autocorrelation in annual rainfall between year of birth and second year before birth among the sampled individuals. HiP: Spearman rank correlation: P = 0.17, but P = 0.0045 when excluding six outlier samples (grey, 3 data points, 3.7% of all samples). KNP: Spearman rank correlation: P = 0.23, but P = 0.047 when rainfall in year of birth ≤ 348 mm/year is excluded (14% of all samples). A large fraction (86–96%) of the samples is characterized by a negative autocorrelation, which can affect the resulting P values in the logistic regression model, i.e., giving significant values in the year of birth rather than the second year before birth. (0.33 MB TIF) [file pone.0001086.s004.tif]
